# Effect of Working After Retirement on the Mental Health of Older People: Evidence From China

**DOI:** 10.3389/fpsyt.2021.731378

**Published:** 2021-09-28

**Authors:** Lin Xie, Yi-dan Yao, Li-li Tang, Shuo Zhang, Hua-lei Yang, Si-qing Zhang, Yuan-yang Wu, Zhi-yun Li

**Affiliations:** ^1^Institution of Population and Labor Economics, University of Chinese Academy of Social Science, Beijing, China; ^2^School of Public Administration, Zhongnan University of Economics and Law, Wuhan, China; ^3^College of Chemistry and Chemical Engineering, Yantai University, Yantai, China; ^4^College of Politics and Public Administration, Qingdao University, Qingdao, China

**Keywords:** re-employment, retirement, mental health, retirement age increase, mediating effect

## Abstract

There is little empirical research on the effect of working after retirement on the mental health of the older adults in China. To fill this gap in the literature, this study examines the effects of working after retirement on the mental health of the older adults using data from the China Family Panel Studies. We employed the methods of ordinary least squares, ordered logit, and propensity score matching–difference in differences (PSM–DID). Results show that working after retirement is negatively related to mental health of the older adults in China. The deterioration effect of post-retirement work mainly impacts those aged over 60 years, women, and those with lower education background, urban household registration, higher pension, and higher social status. Working after retirement is negatively related to mental health through the mediating effects of deteriorating interpersonal relationships and lower positive attitude. It is necessary to consider mental health effects and their population differences to evaluate the impact and improve the quality of policies of active aging.

## Introduction

As the millennial generation of people born after the 1990s has been entering the labor market, the baby-boomers born in the 1960s have been reaching retirement age and withdrawing from the labor market. As a result, the scale of the working-age population is gradually decreasing, the burden of pension increasing, and the problem of aging becoming increasingly serious. According to data from China's National Bureau of Statistics, by the end of 2020, 264 million Chinese were aged 60 years and above, accounting for 18.7% of the total population—an increase of 5.38% from 2010. Furthermore, there were 190.64 million people aged 65 years and above, an increase of 4.63% from 2010. According to the United Nations (1), the proportion of the population aged 60 and above in China will reach 30–40% by 2050, when China will have the largest number of older people among all countries and the fastest aging level. In this context, encouraging the older adults to extend their working life and improving their labor participation rate have become an inevitable choice for China, through active aging measures and delayed retirement policy. Then whether and how continuing to work after retirement affects the older adults' mental health is the questions we are interested in. The answers are related not only to the welfare of the older adults, but also to the development of human resources for this subpopulation and the implementation of a strategy of active aging, healthy China, and a better quality of life.

Some studies have found that working in old age has a positive impact on mental health (2–4). These studies have shown that the underlying mechanism is that work is a symbol of personal identity and status, and that leaving the labor market means losing identity or lowered status, which may reduce the level of mental health. Moreover, the social support theory holds that opportunities for social participation and the social support level of the older adults are likely to decline after retirement, leading to adverse effects on their health. On the one hand, extending working life and thereby increasing social participation and social support may lead to better mental health of the older adults after retirement (3, 5, 6). On the other hand, continuing to work in later years enables many older people to continue to work as they did in middle age, which helps to maintain their sense of meaning and goals in life, and thus, improves the level of their mental health (7, 8) and lowers their mortality rate, especially for those who engage in paid work (9, 10).

However, some studies have found that work may cause the mental health of the older adults to deteriorate, and retirement helps to improve their physical and mental health. The main reasons are as follows. First, the older adults have more flexible use of time after retirement, and they are free to engage in activities besides work, such as exercise and volunteer service, which could improve their health status (11–13). Second, retirement relieves the pressure of work, and living a comfortable life is conducive to the physical and psychological health of the older adults (14, 15). In other words, old age is a time for people to live as they please. The older adults cannot continue to work in the same position after retirement but they may be forced to find other work, which would worsen their physical and psychological health.

Although there are many studies on the relationship between older adults' work and mental health, there are no consensus, and few have examined this topic in China. China's situation is unique, because retirement in China has some degree of mandatory characteristics. In-depth research is required on how re-employment after retirement affects the mental health of the older adults. A few studies, such as Cheng et al. (16), which is based on data from Ningbo City, have found that re-employment after retirement has a positive impact on the mental health of the older adults. However, Huang and Yu (17) found that the mental health level of retired people did not change significantly because of continuing to work. There are no consistent conclusions. Furthermore, these studies either are based on regional samples or do not conduct in-depth analysis of the impact mechanism and do not consider the endogeneity problem. To fill these research gaps, this study empirically tests the mental health effect of working after retirement on the older adults and conducts an in-depth analysis of its impact mechanism.

On the basis of the foregoing, we put forward the three competing hypotheses as follows:

Hypothesis 1a: Working after retirement has no significant association with depressive symptoms in older adults.

Hypothesis 1b: Working after retirement will worsen the depressive symptoms of older adults.

Hypothesis 1c: Working after retirement will benefit the depressive symptoms of older adults.

Some studies have found that the impact of working after retirement on psychological well-being was heterogeneous in terms of individual characteristics (18, 19). Moreover, analysis of the effects on different groups is necessary. Therefore, the following hypothesis can be proposed:

Hypothesis 2: The impact of working after retirement on the depressive symptoms of older adults is heterogeneous in different groups.

Further, we would like to explore the mechanism behind the relationship between reemployment and the mental health of older adults. According to activity theory and studies on retirement, work may help the elderly increase income, increase the opportunities for interpersonal communication (3, 5, 20), and maintain a positive attitude, which is conducive to one's mental health. Thus, we hypothesize the following:

Hypothesis 3: Working after retirement will affect the depressive symptoms of older adults by affecting their financial income, interpersonal relationships, and self-rated confidence in the future.

## Methods

### Data

The data used in this study are from the CFPS in 2018. The CFPS is a large-scale survey conducted by the Social Survey Center of Peking University. A multi-stage probability proportional to size strategy with implicit stratification was performed in the sampling process that comprises three stages: county level as the primary sampling unit, a community or village for the second-stage sampling unit, and household as the final sampling unit (21). The survey covers 25 provinces, municipalities, and autonomous regions, representing 95% of the population on the Chinese mainland. In 2018, the survey included 29,478 adults. The data contain rich information about the retirement, work, and mental state of older adults, which is suitable for the analysis in this study.

We carry out a series of processing to the original data. First, the sample is limited to urban respondents who have gone through retirement procedures or have received pensions. Second, observation values of those aged <45 years and over 80 years are excluded from the sample construction. The reasons are as follows. First, the legal minimum retirement age in China is 50 years for women, and the policy stipulates that a person may retire 5 years before the minimum legal age; thus, samples aged under 45 years should be excluded. Second, the proportion of people aged over 80 years who work is very small, and the level of change is miniscule. Finally, observations whose main variables are missing are deleted. The number of deleted observations is 46. The final study sample consists of 3,940 observations.

### Variables

#### Dependent Variable

The dependent variable is depressive symptoms. The CFPS questionnaire includes the Center for Epidemiologic Studies Depression Scale (CES-D), which is a commonly used international scale for measuring depression (22). According to existing research, this study uses the CES-D to measure depressive symptoms. According to the CES-D scale in the 2018 CFPS data, negative emotions are classified based on answers to the following questions: “I feel depressed,” “It feels very hard to do anything,” “I don't sleep well,” “I feel lonely,” “I feel sad,” and “I can't continue my life.” The answers correspond to four options: almost never (<1 day a week), sometimes (1–2 days), often (3–4 days), and most of the time (5–7 days), which are assigned values of 1, 2, 3, and 4, respectively. “I feel happy” and “I live a happy life” reflect positive emotions, which are assigned in reverse. The total score of the CES-D ranges between 8 and 32. The higher the score, the worse the mental health, and vice versa.

#### Independent Variable

The core independent variable is “working after retirement,” that is, whether the person continues to work or finds new work after retirement. If the respondents have gone through the retirement procedures but still work, the variable is set as 1, whereas if the respondents have retired and quit the labor market, it is set as 0.

#### Covariates

All regression specifications are adjusted for several covariates that may confound the estimates of the effect of work after retirement on mental health. This study selects the individual characteristics, family characteristics and socio-economic characteristics of the respondents as control variables. The variables for individual characteristics include age, gender, household registration (*hukou*), years of education, marital status, and health status. The variables for family characteristics include family care and total number of families. The variables for socio-economic characteristics include receiving pension status, the logarithm of family per capita income, and social status.

#### Mediating Variables

If re-employment after retirement has a significant impact on the mental health of the older adults, then we need to clarify the reasons. First, work may help the older adults to increase their income. Second, according to activity theory, work may increase opportunities for interpersonal communication and help to maintain a positive attitude (20), thereby improving the health of the older adults. Based on previous related research, this study examines the mechanism of re-employment after retirement on mental health through the three aspects of financial income, interpersonal communication, and positive attitude. Financial income is measured by income self-evaluation, interpersonal relationships are measured by self-rated interpersonal relationships, and positive attitude is measured by whether the respondents have confidence in the future. Self-evaluation, self-rated interpersonal relationships, and confidence in the future all correspond to answers in the questionnaire. The respondents provide a self-evaluation using a maximum of five points and a minimum of one point. Table 1 provides the definitions of the variables.

**Table 1:** Variables and definitions.

**Variables**	**Definitions**
**Primary variables**	
*CESD*	Depressive symptoms, which is measured by the depression score obtained by the CES-D scale in the CFPS, with a value range of 8–32. The higher the score, the more obvious the depressive symptoms.
*Re-employ*	Re-employed after retirement = 1; Retired and not working = 0
**Personal characteristic variables**	
*Age*	Age
*Gender*	Male = 1; Female = 0
*Hukou*	Urban *hukou* = 1; Agricultural *hukou* = 0
*Education*	Years of education: Illiterate = 0; Primary school = 6; Junior high school = 9; Senior high school = 12; Junior college = 15; Undergraduate = 16; Graduate = 18
*Marital status*	Married = 1; Other = 0
*Health*	Good = 3; Mediocre = 2; Bad = 1
**Family characteristic variables**	
*Fml_care*	Do housework for children or take care of grandchild = 1; Other = 0
*Fml_count*	Number of family members
**Socio-economic characteristic variables**	
*Pension*	Above the mean = 1; Under the mean = 0
ln*income*	Logarithm of per capita household income
*Caste*	Self-rated social status, with a value range of 1–5; the higher the value, the higher the social status.
**Mediating variables**	
*IS*	Self-rated income status, with a value range of 1–5. The higher the value, the higher the income status.
*IR*	Self-rated interpersonal relationships, with a value range of 1–5; the higher the value, the better the interpersonal relationships.
*PA*	Self-rated confidence in the future, with a value range of 1–5; the greater the value, the higher the confidence in the future

### Models

#### Basic Model

To empirically examine the effect of working after retirement on the mental health of the older adults, the econometric model is set as follows:


(1) $$\begin{array}{l} CESD_{i}=\alpha_{1}+\alpha_{2}reemploy_{i}+\alpha_{3}X_{i}+\varepsilon_{i} \end{array}$$

where *CESD*_*i*_ is the dependent variable of focus, depressive symptoms. *reemploy*_*i*_ is the independent variable; *X*_*i*_ is a series of control variables, namely, personal demographic characteristics, family characteristics, and socio-economic characteristics; and ε_*i*_ is a random error term. Depressive symptoms can be regarded as a continuous variable. In this study, OLS is employed for the first step in the investigation. Considering that the explained variables are ordinal variables, the ordered logit regression model is also employed. Thus, we can augment the robustness of the estimation results.

#### Treatment of Endogenous Problems: PSM–DID Method

The following endogeneity problems may bias our main results. First, there may be sample selection concerns, that is, the observations for those working after retirement and not working after retirement have heterogeneous initial conditions, as work is not randomly assigned to the retired older adults. Second, there may be unobservable omitted variables that affect both the mental health of the older adults and whether the residents work after retirement. Following Van den Broeck and Maertens (23), we combine the differences-in-differences estimation with propensity score matching (PSM–DID) to overcome these problems and check the robustness of the main results. Therefore, based on the two periods of balanced panel data constructed using CFPS data in 2016 and 2018, the study employs the PSM–DID method to re-estimate the impact of working after retirement on mental health. According to the basic principle of the PSM–DID method, the basic model is


(2) $$\begin{array}{l} ATT_{PSM-DID}=E[(Y_{1}^{T}-Y_{0}^{T})|X_{0},D=1]-\\ \;E[(Y_{1}^{C}-Y_{0}^{C})|X_{0},D=0] \end{array}$$

where *D* is the dummy variable (1 for the treatment group, 0 for the control group), *T* is the treatment group, *C* is the control group, *Y*_0_ is the depression score of the baseline group, *Y*_1_ is the depression score of the control group, and *X* represents the covariates. The key point of PSM–DID is to replace the depression score of cross-sectional data with that of panel data based on the propensity score matching (PSM). This method is similar to the quasi-experimental method, which provides an estimate with less selection bias by creating similar features between the treatment and control groups.

#### Mediating Mechanism Analysis Model

Refer to the mediating effect test method of Wen and Ye (24), Equations (3) and (4) were constructed based on Formula (1).


(3) $$\begin{array}{l} mediator_{i}=\gamma_{0}+\gamma_{1}reemploy_{i}+\gamma_{2}X_{i}+\varepsilon_{i} \end{array}$$

(4) $$\begin{array}{l} CESD_{i}=\delta_{0}+\delta_{1}mediator_{i}+\delta_{2}reemploy_{i}+\delta_{3}X_{i}+\varepsilon_{i} \end{array}$$

Here, *mediator*_*i*_ is the mediating variable. According to the mediating effect test method of Wen and Ye (24), the first step is to test the influence of working after retirement on depressive symptoms, that is, the coefficient *α*_2_ of Formula (1). If *α*_2_ is significant, it indicates a mediating effect, and otherwise, a masking effect; The second step is to test *γ*_1_ and *δ*_1_ in turn. If all of them are significant, the mediating effect is significant; if at least one is not significant, we continue to use bootstrap and other methods for the test. The third step is to observe whether *δ*_2_ is significant. If it is not, then the direct effect is not significant, indicating that there is only an intermediary effect but no direct effect; if *δ*_2_ is significant, there is a partial mediating effect. A masking effect is indicated if *γ*_1_ and *δ*_1_ have different signs. A partial mediating effect is indicated by the magnitude of the effect |*γ*_1_·*δ*_1_/*α*_2_|.

## Results

### Descriptive Analysis of Variables

Table 2 presents the descriptive statistics of the main variables of the sample. On the whole, the depression scores of the whole sample, retired without work, and working after retirement are all observed at low levels; overall, the mental health status is good. In the entire sample, those who were re-employed after retirement accounted for 38% of the total sample, 76% of the total sample were aged 60 or over, and 45% of the sample were male, and 61% of the total sample was urban residence. The mean years of education in the entire sample was 7.3 years. Forty-four percent of older people had junior secondary education or less. The proportion of those who were married was approximately 85%. Seventy-five percent of older people provide home care for their children and 54% had an above average pension income.

**Table 2:** Descriptive statistics of the variables.

**Variables**	**Full sample**			**Re-employ** **=** **0**			**Re-employ** **=** **1**			***t*** **-test**	
	***N***	**mean**	**sd**	***N***	**mean**	**sd**	***N***	**mean**	**sd**	***t*** **-value**	***P*** **(|***T***| > |***t***|)**
*CESD*	3,940	12.980	4.083	2,438	12.842	4.076	1,502	13.202	4.086	−0.360^***^	0.007
*Re-employ*	3,940	0.381	0.486	2,438	0	0	1,502	1	0	—	—
*Age*	3,940	64.503	7.574	2,438	65.922	7.286	1,502	62.200	7.469	3.723^***^	0.000
45–60	3,940	0.236	0.425	2,438	0.182	0.386	1,502	0.324	0.468	−0.142^***^	0.000
60–80	3,940	0.764	0.425	2,438	0.818	0.386	1,502	0.676	0.468	0.142^***^	0.000
*Gender*	3,940	0.445	0.497	2,438	0.408	0.492	1,502	0.507	0.500	−0.099^***^	0.000
*Hukou*	3,934	0.606	0.489	2,433	0.755	0.430	1,501	0.364	0.481	0.390^***^	0.000
*Education*	3,893	2.589	1.076	2,403	2.728	1.057	1,490	2.365	1.070	1.473^***^	0.000
Illiterate	3,893	0.215	0.411	2,403	0.177	0.382	1,490	0.277	0.447	−0.099^***^	0.000
Primary school	3,893	0.223	0.416	2,403	0.199	0.400	1,490	0.261	0.439	−0.062^***^	0.000
Middle school	3893	0.319	0.466	2,403	0.341	0.474	1,490	0.283	0.451	0.058^***^	0.000
High school or more	3893	0.243	0.429	2,403	0.282	0.450	1,490	0.179	0.384	0.103^***^	0.000
*Marital status*	3940	0.853	0.354	2,438	0.83	0.376	1,502	0.891	0.311	−0.062^***^	0.000
*Health*	3,940	2.352	0.828	2,438	2.294	0.847	1,502	2.445	0.788	−0.151^***^	0.000
*Fml_care*	3,940	0.371	0.483	2,438	0.388	0.487	1,502	0.342	0.475	0.046^***^	0.004
*Fml_count*	3,924	3.577	1.896	2,424	3.398	1.846	1,500	3.866	1.941	−0.468^***^	0.000
*Pension*	3,939	0.540	0.498	2,437	0.703	0.457	1,502	0.276	0.447	0.427^***^	0.000
Lnincome	3,858	7.850	3.563	2,407	7.338	4.059	1,451	8.700	2.294	−1.362^***^	0.000
*Caste*	3,925	3.211	1.097	2,428	3.149	1.081	1,497	3.311	1.116	−0.162^***^	0.000
*IS*	3,837	2.966	1.111	2,357	2.914	1.090	1,480	3.048	1.139	−0.134^***^	0.000
*IR*	3,934	7.309	1.985	2,436	7.326	1.967	1,498	7.282	2.016	0.044	0.504
*PA*	3,935	4.108	1.004	2,434	4.089	1.014	1,501	4.139	0.986	−0.049	0.134

*, **, and *** *indicates significance level at 1%, 5% and 10%, respectively*.

Comparing the retired re-employed group with the retired not re-employed group, we found that older people who withdrew from the labor market after retirement had significantly lower mean depression scores. In addition, the proportion of men who retired and re-employed is higher those who retired and did not re-employ (50.7%); the proportion of agricultural *hukou* (household registration) is higher (67%); and the education level is lower (about 6.4 years on average). There exists a higher proportion of people with lower than average pension. However, those who are re-employed after retirement have better health, higher per capita family income, and higher social status. They are younger (average age of 62 years), represent a higher proportion of married people, and seldom provide family care for their children. Generally, those working after retirement have better social, financial, and physical status than those who are retired and not working; however, their mental health is poorer than the latter.

### Basic Results Analysis

Table 3 reports the estimation results after adding different control variables. The OLS and ordered logit regression of models 1 and 2 do not include any control variables, models 3 and 4 add variables for individual characteristics, and models 5 and 6 add variables for family characteristics and socio-economic characteristics. As shown in Table 3, working after retirement has a negative association with the older adults' mental health. After controlling individual characteristics, family characteristics, and socio-economic characteristics, model 5 shows that re-employment after retirement significantly increases the depression score of the older adults by 0.382. These results are consistent with the results from the descriptive statistics shown in Table 2. Although the estimations indicate that working after retirement adversely impacts older adults' mental health, the estimated effect might still be biased because the working after retirement is likely to be selected and endogenous with mental health. These findings should be viewed alongside further robustness checks described below.

**Table 3:** Results of OLS and ordered logit on effects of re-employment after retirement on mental health.

**Variables**	**(1)**	**(2)**	**(3)**	**(4)**	**(5)**	**(6)**
	**OLS**	**Ordered Logit**	**OLS**	**Ordered Logit**	**OLS**	**Ordered Logit**
*Re-employ*	0.262^*^	1.143^**^	0.308^**^	1.163^**^	0.382^***^	1.203^***^
	(0.137)	(0.068)	(0.145)	(0.080)	(0.147)	(0.085)
*Age*			−0.017^*^	0.989^***^	−0.007	0.993
			(0.009)	(0.004)	(0.010)	(0.005)
*Gender*			−0.882^***^	0.610^***^	−0.910^***^	0.597^***^
			(0.127)	(0.037)	(0.128)	(0.037)
*Hukou*			−0.203	0.919	−0.306^*^	0.871
			(0.173)	(0.076)	(0.176)	(0.075)
*Education (Illiterate = 0)*						
Primary school			−0.280	−0.922	−0.277	0.925
			(0.184)	(0.081)	(0.186)	(0.083)
Middle school			−0.464^**^	−0.819^**^	−0.539^***^	0.791^**^
			(0.188)	(0.074)	(0.190)	(0.073)
High school or more			−0.678^***^	0.777^**^	−0.742^***^	0.754
			(0.209)	(0.078)	(0.212)	(0.077)
*Marital status*			−1.464^***^	0.535^***^	−1.410^***^	0.542^***^
			(0.175)	(0.046)	(0.176)	(0.047)
*Health status (poor = 0)*						
Mediocre			−1.991^***^	0.439^***^	−1.943^***^	0.451^***^
			(0.187)	(0.039)	(0.189)	(0.041)
Good			−3.270^***^	0.219^***^	−3.141^***^	0.232^***^
			(0.149)	(0.016)	(0.151)	(0.018)
*Pension*			−0.613^***^	0.729^***^	−0.566^***^	0.747^***^
			(0.165)	(0.057)	(0.167)	(0.061)
*Fml_care*					−0.112	0.971
					(0.132)	(0.061)
*Fml_count*					−0.043	0.974
					(0.036)	(0.017)
*ln income*					0.003	1.002
					(0.018)	(0.009)
*Caste*					0.438^***^	0.817^***^
					(0.056)	(0.022)
Province	Yes	Yes	Yes	Yes	Yes	Yes
*N*	3,940	3,940	3,886	3,886	3,777	3,777
*R^2^*	0.036	0.007	0.204	0.041	0.217	0.044

*Ordered logit results are odds ratios; those in brackets are robust standard errors*.

*, **, and *** *indicates significance level at 1%, 5% and 10%, respectively*.

Apart from working after retirement, several sociodemographic variables affect mental health of the older adult as well. Men are likely to have better mental health status than women. And as expected, mental health of older adults with higher education are better than those with lower education. Older adults with normal marriage are less depressed than the divorced or widowed. Older adults with urban Hukou have better mental health status than those in agricultural Hukou. Those who are healthier, have higher pension and higher social status tend to have better mental health status.

### Robustness Analysis: PSM–DID Estimation Results

Considering there may be endogeneity problems, such as sample selection and omitted confounding variables between the retired and mental health, this study employs PSM–DID for robustness checks. In this subsection, we construct two-period panel data from the CFPS for the years 2016 and 2018. The retired who newly joined the workforce in the 2018 wave—that is, the retired who did not work in the 2016 wave but worked in the 2018 wave—are taken as the treatment group. The retired who worked neither in 2016 nor in 2018 are taken as the control group. After corresponding processing of the data, we have 177 observations of the treatment group and 1,497 observations of the control group. Then, we estimate the propensity score of work of the retired through a binary logit regression model, including the individual characteristics, family characteristics, and socio-economic characteristics, and we match samples according to the propensity score.

**Figure 1:**
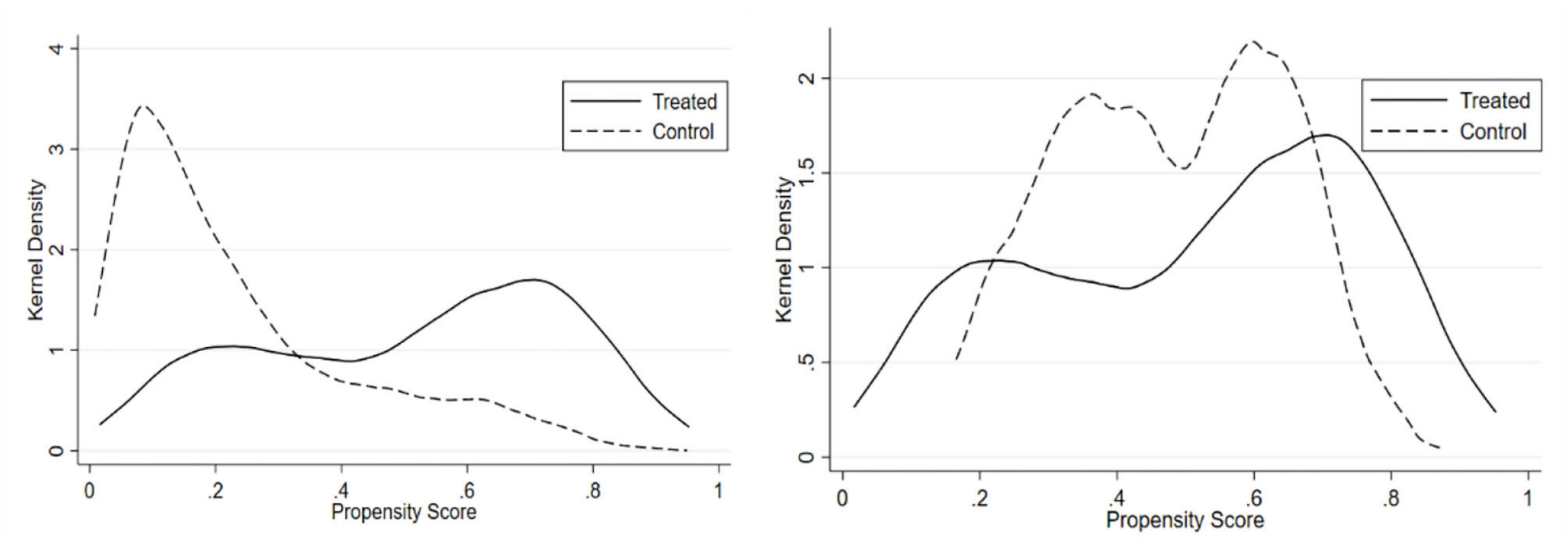
Propensity distribution of the treated and control groups before (left) and after (right) matching.

To ensure the validity of the PSM–DID method, we conduct additional analyses. Figure 1 shows the propensity distribution of the treated and control groups before and after matching. The results demonstrate a noteworthy extension of the common support between the treated and the control groups, implying that the overall distributions of the conditional probability to return to work are similar between the two groups. Furthermore, we check whether the data are balanced. Table 4 presents the results of covariates balance testing for PSM before and after matching. Although there are significant differences in some variables between the unmatched treatment and the control groups, the differences of all variables are no longer significant after matching, implying that the matching effect is great.

**Table 4:** Results of PSM data balanced test.

	**Unmatched**	**Mean**			**Percentage of reduction (%)**	***t*** **-test**	
**Variable**	**Matched**	**Treated**	**Control**	**%bias**	**|bias|**	***t***	***p*** **>** ***t***
*Age*	U	63.783	65.846	−30.9		−10.04	0.000
	M	63.829	64.117	−4.3	86.1	−1.23	0.217
*Gender*	U	0.491	0.409	16.5		5.39	0.000
	M	0.489	0.485	0.7	95.7	0.20	0.844
*Hukou*	U	0.370	0.809	−99.7		−33.76	0.000
	M	0.371	0.364	1.6	98.4	0.41	0.683
*Education*	U	5.710	7.748	−44.2		−14.54	0.000
	M	5.717	5.542	3.8	91.4	1.04	0.300
*Marital status*	U	0.883	0.845	11		3.51	0.000
	M	0.882	0.880	0.6	94.9	0.17	0.868
*Health*	U	2.409	2.284	15.5		4.99	0.000
	M	2.408	2.372	4.6	70.4	1.29	0.196
*Fml_care*	U	0.367	0.405	−7.7		−2.50	0.012
	M	0.369	0.357	2.5	67.9	0.70	0.484
*Fml_count*	U	3.803	3.375	23.1		7.68	0.000
	M	3.800	3.807	−0.4	98.4	−0.09	0.925
ln*income*	U	8.902	8.469	15.7		4.74	0.000
	M	8.902	8.903	−0.1	99.7	−0.02	0.985
*Pension*	U	0.329	0.740	−90.6		−29.92	0.000
	M	0.330	0.324	1.1	98.8	0.30	0.760
*Caste*	U	3.166	3.006	14.4		4.72	0.000
	M	3.164	3.224	−5.4	62.5	−1.45	0.147
Sample	*P*s *R*^2^	LR chi^2^	*p* > chi^2^	MeanBias	MedBias	B	R
Unmatched	0.239	1464.17	0	33.6	16.5	130.1^*^	1.12
Matched	0.002	6.95	0.803	2.3	1.6	9.4	0.95

*B represents the absolute standard deviation and R denotes the standard deviation ratio*.

***, ** and * *indicates significance level at 1%, 5% and 10%, respectively*.

Table 5 shows the results estimated by applying the PSM–DID method. Table 5 shows that in 2016, there was no significant difference in mental health status between the treatment group (the older adults working after retirement) and the control group (the retired and not working); meanwhile, the mental health status of the retired older adults in the treatment group was better than that in the control group, with the average depression score about 0.174 lower than that for the control group. However, in 2018, after the retired in the treatment group returned to work, their depression scores were significantly higher than those in the control group by an average of about 0.306. Subtracting the difference between the treatment group and the control group in 2016 and 2018, we find that the average treatment effect of the treatment group is 0.479, which is significant at the 10% level. Thus, the results support Hypothesis 1b and reject Hypotheses 1a and 1c. This indicates that working after retirement causes the mental health of the older adults to deteriorate, which echoes the baseline result estimated by OLS and ordered logit and confirms the robustness of the results to a certain degree.

**Table 5:** Results estimated by PSM–DID.

	**Baseline diff**	**Follow-up diff**	**Diff-diff**
Diff value	−0.174	0.306^*^	0.479^*^
Standard deviation	0.168	0.183	0.248
*t*-value	−1.03	1.67	1.93
*p*-value	0.301	0.095	0.054

*, **, and *** *indicates significance level at 1%, 5% and 10%, respectively*.

### Effects by Sub Groups

Analysis of the effect on different groups would provide a reference for more accurate policy intervention. The sample is stratified by age, gender, education background, *hukou*, pension level, and social status. The results are reported in Table 6.

**Table 6:** Effects of stratified sample (OLS and OLogit estimation).

	**Age**				**Gender**			
	**60** **<** **age** **<** **80**		**45** **≤** **age** **≤** **60**		**Female**		**Male**	
*Re-employ*	0.399^**^	1.207^**^	0.232	1.140	0.592^***^	1.322^***^	0.109	1.101
	(0.176)	(0. 099)	(0.264)	(0. 158)	(0.193)	(0.119)	(0.214)	(0.118)
Control variables	Yes	Yes	Yes	Yes	Yes	Yes	Yes	Yes
*R^2^*	0.227		0.2223		0.205		0.215	
*N*	2,890	2,890	891	887	2,090	2,090	1,687	1,687
	**Education level**				**Hukou**			
	**Under the mean**		**Above the mean**		**Agricultural household**		**Non-agricultural household**	
*Re-employ*	0.605^***^	1.322^***^	0.182	1.123	0.317	1.195^*^	0.455^**^	1.206^*^
	(0.226)	(0. 133)	(0.191)	(0. 110)	(0.231)	(0.124)	(0.194)	(0.118)
Control variables	Yes	Yes	Yes	Yes	Yes	Yes	Yes	Yes
*R^2^*	0.211		0.211		0.233		0.202	
*N*	1,699	1,699	2,123	2,123	1,490	1,490	2,301	2,287
	**Pension**				**Social status**			
	**Under the mean**		**Above the mean**		**Low social status**		**High social status**	
*Re-employ*	0.304	1.156	0.398^*^	1.215^*^	0.240	1.158	0.567^**^	1.285^**^
	(0.216)	(0.112)	(0.205)	(0.128)	(0.359)	(0.177)	(0.231)	(0.148)
Control variables	Yes	Yes	Yes	Yes	Yes	Yes	Yes	Yes
*R^2^*	0.215		0.207		0.215		0.228	
*N*	1,743	1,743	2,034	2,034	814	814	1,339	1,339

*, **, and *** *indicates significance level at 1%, 5% and 10%, respectively. Standard errors are presented in parentheses*.

The estimation results show that the estimated coefficient of age in the younger group (45–60 years) is 0.232, which is not significant, while it is 0.399 in the older group (60–80 years) and significant at the 5% level. This means that re-employment after retirement has no significant effect on the mental health of the younger group, but significantly worsens the mental health status of the older group. From the perspective of gender, women mainly experience adverse effects on mental health from working after retirement. The estimated coefficient of the female group is 0.592, significant at the 1% level, while the coefficient of the male group is not significant. From the perspective of education level, the detrimental effects of working after retirement on the mental health of the older adults mainly affects the group with lower education background. We divide the sample into a lower education group and a higher education group split by average education years and conduct linear model estimation on each. The estimated coefficient for the lower education group is 0.605, significant at the 1% level, but the influence coefficient of the higher education group is not significant. Surprisingly, we find that the detrimental effects of re-employment after retirement on the mental health of the older adults mainly affects the urban group, the higher pension group, and the higher social status group. Overall, the results are consistent with Hypothesis 2, that is, the impact of working after retirement on mental health is heterogeneous in terms of individual characteristics.

### Mediating Mechanism Analysis

We confirm that working after retirement significantly affects the mental health status of retired older adults. However, by what channels does this effect take place? Work can help the older adults to increase their income. According to activity theory, work can also increase opportunities of interpersonal communication and help to maintain a positive attitude, so as to improve the health of the older adults. Therefore, this study examines the mediating mechanism of working after retirement on mental health through the three aspects of financial income, interpersonal relationships, and positive mental attitude.

Table 7 reports the overall regression results. Column 1 shows the result without any intermediary variable; columns 2, 4, and 6 show the regression results of the independent variable to each of the three intermediary variables; and columns 3, 5, and 7 add the regression results of economic income status, interpersonal relationships, and positive mental attitude, respectively. The results show that working after retirement significantly improves the level of financial income, while it worsens interpersonal relationships and has a negative impact on positive attitude, albeit not significant. The final test results show that the three factors have different effects on mental health. The results are consistent with Hypothesis 3. Income status has a 0.6% masking effect, while interpersonal relationships and positive attitude explain more than 5% of the mediating effect, especially positive mental attitude. After adding this variable, the influence of post-retirement work on mental health is significantly reduced.

**Table 7:** Results of mediating mechanism testing.

	**(1)**	**(2)**	**(3)**	**(4)**	**(5)**	**(6)**	**(7)**
	**CESD**	**IS**	**CESD**	**IR**	**CESD**	**PA**	**CESD**
*Re-employ*	0.382^***^	0.136^***^	0.354^**^	−0.014	0.372^**^	−0.017	0.365^**^
	(0.147)	(0.039)	(0.149)	(0.076)	(0.146)	(0.037)	(0.141)
*IS*			−0.017				
			(0.063)				
*IR*					−0.154^***^		
					(0.031)		
*PA*							−1.028^***^
							(0.062)
Covariables	Yes	Yes	Yes	Yes	Yes	Yes	Yes
*Province*	Yes	Yes	Yes	Yes	Yes	Yes	Yes
*N*	3,777	3,685	3,685	3,774	3,774	3,775	3,775
*R* ^2^	0.217	0.272	0.221	0.100	0.221	0.171	0.269

***, **, * * indicates significance level at 1%, 5% and 10%, respectively. Standard errors are presented in parentheses. The mediating effect bootstrap test passed the 5% significance level test*.

## Discussion

### Working After Retirement and Mental Health Are Negatively Correlated

Overall, both the basic results and the results obtained from the robustness test show that working after retirement increases the depression score of older adults and aggravates their mental health, supporting Hypothesis 1b. This could be significantly related to the context of China. First, the driving force of working after retirement is more of a “push” factor than a “pull” factor in China. Factors affecting retired older adults' willingness to work can be divided into four categories: financial needs, work needs, giving full play to their strengths, and spiritual sustenance (25). Financial needs can be regarded as push factors, while the other three factors can be considered as pull factors. However, in China, financial factors are the main drivers of the re-employment of retired older adults (20, 26). It is not easy to improve the mental health of older adults based on a push factor as the motivation to work. Second, China has had a mandatory retirement policy for a long time. This greatly affects the employment prospects of older people. For Chinese older people, it is widely believed that retirement is a time when older people should enjoy life. Older people returning to employment after retirement may be perceived as a result of ungrateful children or family misfortune, which puts enormous psychological pressure on older people. Moreover, the long-term implementation of the mandatory retirement policy has also caused most elderly individuals to face a variety of discrimination with regard to demotions and reductions in wages and benefits from their employers (27), which are also detrimental to the mental health of the elderly. Moreover, those individuals who are re-employed after retirement face a lot of work pressure, which negatively affects their mental health. According to the disengaging theory, older adults should be released from social activities in their later years. Maddox (28) and Moen (29) further pointed out that quitting work could relieve stress and thus be beneficial to physical and mental health. However, when older adults re-enter the labor market, there are work responsibilities which are not conducive to their mental health.

### Heterogeneous Impact of Working After Retirement on Mental Health

Although the relationship between working after retirement and depression among the older adults was regulated by group differences, the empirical results supports hypothesis 2. In different age groups, working after retirement has no significant effect on the mental health of the younger group, but it significantly worsens the mental health status of the older group. Carstensen et al. (30) explained this from the perspective of social emotional choice theory. They considered that with age, the older adults perceive future time as limited, and they gradually become more willing to focus on their inner circle of social network relationships, such that they pay more attention to intimate relationships. Under the cultural background of China, retirees are expected to be cared for by their grandchildren and enjoy the happiness associated with being with their family. This concept is deeply rooted in the hearts of the Chinese people, especially among older women. Thus, older Chinese individuals pay more attention to leisure time outside of work, such that the benefit of leisure is higher than that of work (20). There is an increasing conflict between the desire to continue this tradition and the reality of having to work after retirement. Therefore, the deteriorating effect of mental health is more obvious for the cohort of older adults. Thus, we should pay more attention to the crowding out of mental health welfare of older adults when implementing a policy for increasing retirement age.

Such a difference also existed among between genders and education levels. Women experienced adverse effects on their mental health from working after retirement since older women are more expected to enjoy the happiness associated with family. Moreover, women are conflicted in their role in terms of providing family care and working due to the tradition thinking of “men work outside their home, women work inside their home.” Van Houtven et al. (31) found that female workers who continued to work after retirement reduced their working hours and wages because they were required to care for their families; however, this had little impact on men. In the Chinese cultural background, men dominate the work outside their home, while women dominate work at home; China's current three-child policy implies that retired women assume more family care. These conditions exacerbate the conflict between family responsibilities and work faced by women who work after retirement; this worsens their mental health. From the perspective of education, the detrimental effects of working after retirement on the mental health of older adults affects the group with a lower education background. According to Yang et al. (32), among low education groups, income compensation drives the return to work, that is, they re-enter the labor market because of financial needs, which is not conducive to mental health.

Finally, the detrimental effects on the mental health of working after retirement of older adults mainly affect the urban, higher pension, and higher social status groups. This seems to contradict the analysis of the push–pull theory introduced earlier in this section. However, with careful consideration, we think that the level of pension is generally positively related to the level of social status, and people with higher social status usually have higher pensions after retirement. Generally, people with higher social status have stronger abilities than those with a lower social status and have greater labor supply elasticity, such that they are more likely to work after retirement because of the presence of pull forces. However, in reality, their authority after re-employment is often greatly reduced, leading to a large psychological expectation gap, which is not conducive to their mental health.

### Mediating Mechanism

Results show that financial income, interpersonal relationships, and positive mental attitude have different effects on mental health. However, the positive effects of work contributed by income cannot offset the negative effects contributed by the latter two.

Specifically, re-employment after retirement has a significant positive boost to older people’s financial income, which is positively related to older people’s mental health. This is consistent with the findings of mainstream research, where in general, higher income earners generate more positive emotions and lower income earners suffer more negative emotions (33). In this study, though work is generally regarded as an important form of social participation in the elderly, it is negatively related to interpersonal communication and positive attitudes—although not significant—and through them, it significantly and negatively affects mental health. The findings are contrary to the conclusions derived by Kim and Moen (3) and Forbes et al. (6). This can be explained as follows. Because of their perceived limited lifespan, older people are willing to invest more time in intimate relationships (30), especially in China, a country that has long been influenced by Confucian culture. Moreover, China's current retirement age system and cultural norms are not conducive to the active employment of older adults due to which older people undergo further discrimination in employment. Thus, the interpersonal relationships of the retired who continue to work have not been significantly improved; on the contrary, there may be a trend of deterioration, and it has a negative impact on their positive attitudes. Therefore, when the elderly evaluate the quality of interpersonal relationships and positive attitudes, it is seen that post retirement work has no advantage.

## Conclusion

Using data from the CFPS, this study examined the impact of re-employment on the mental health of older adults who retired in China. We found that re-employment significantly increased older people's depression scores and worsened their mental health. In addition, the impact of re-employment on older people's mental health may depend on their socio-economic background, which mainly affects older people in the sample who are over 60 years of age, female by gender, and those with lower educational background, urban residents, higher pensions and higher social status. We also found that income status had a 0.6% masking effect on mental health in the interaction between re-employment and mental health. Interpersonal relationships and positive attitudes explained more than 5% of the mediating effect, especially positive attitudes. The effect of re-employment on mental health was significantly reduced by the inclusion of this variable.

The theoretical contribution of this study is that it enriches the existing research on the impact of extending working life and mental health by considering the Chinese cultural background. We not only compare the differences between the mental health of the older adults who work after retirement and those who do not, but also discuss the heterogeneity and mediating effects so as to further clarify the influence mechanism between working after retirement and the mental health of older adults people. Although re-employment can address aging problems from a macro perspective and improve the financial income of the older adults at a micro level, we should also pay attention to its possible adverse effects on their mental health.

The findings in this paper are specific to the Chinese context. The impact of re-employment on mental health may be influenced by economic conditions and cultural background. So, whether our results are applicable to other countries deserves further study. A limitation of this study is that multiple comparisons were not conducted. Although we can conclude that re-employment in China somewhat leads to deterioration in mental health among older cohorts, and a number of robustness tests, including the PSM-DID strategy, OLS models and OLogit models, support our findings, these estimates are still subject to some limitations. Additional research using other data sources and methods would help to further strengthen our findings.

In spite of the limitations, this study has important implications for active aging in China and other developing countries. Against the current background of aging populations, several countries have implemented or are implementing delayed retirement programs, and this has been the inevitable choice for China too. The social policy of raising the retirement age will help society to develop a better concept of promoting the employment of older people. However, when implementing a plan to increase retirement age, the following three aspects should be considered, based on the findings of this study. First, there should be a gender-specific retirement policy or flexible retirement policy. A policy to delay retirement age needs to pay attention to work–family balance. Especially in East Asian countries, the family division of labor is still male dominated for outside work and female dominated for work in the home. Women undertake more family care work, and the cost of delayed retirement for women is higher than that for men. Second, retirement policy should aim to improve the replacement rate of personal pension and to increase the income level from older adults employment. Most people who work after retirement do so because they need to earn income. Increasing their income level could improve the welfare of the employed older adults to some extent and thus, alleviate the adverse effects of delayed retirement. Third, it is necessary to improve the re-employment environment of the retired older adults and to enhance their social status of continuing employment. China should strengthen legislation and implement anti-age discrimination measures, ensure that older adults workers enjoy equal opportunities in human resource management, build older adults friendly workplaces, create a good, friendly working atmosphere for the older adults, and achieve a positive aging experience for the older adults. Finally, China should actively advocate policies for living longer, working longer, and lifelong learning to change the traditional concept of stopping work after retirement. This would gradually enhance the society's identification of working until later life at a conceptual level and stimulate the enthusiasm of the older adults to participate in productive work.

## Data Availability Statement

Publicly available datasets were analyzed in this study. The datasets for this study are available from the CFPS at http://www.isss.pku.edu.cn/cfps/index.htm?CSRFT=PRT1-FZE8-0ANZ-O2DR-A76M-3PTJ-AH2S-7UKF.

## Author Contributions

LX and L-lT conceived this research. LX, L-lT, S-qZ, and SZ was responsible for the methodology and conducted software analyses. Y-dY and L-lT conducted necessary validations. Y-yW and L-lT conducted a formal analysis and managed the investigation. L-lT and Y-dY gathered resources, curated all data, wrote/prepared the original draft, and were responsible for project administration. LX and L-lT reviewed and edited the manuscript, were responsible for visualization, supervised the project. H-lY and Z-yL acquired funding. All authors contributed to the article and approved the submitted version.

## Funding

This study was supported by the Humanities and Social Sciences Fund of the Ministry of Education (Grant No. 19YJC790167) and the Social Science Fund of Shandong Province of China (Grant No. 21DRKJ01).

## Conflict of Interest

The authors declare that the research was conducted in the absence of any commercial or financial relationships that could be construed as a potential conflict of interest.

## Publisher's Note

All claims expressed in this article are solely those of the authors and do not necessarily represent those of their affiliated organizations, or those of the publisher, the editors and the reviewers. Any product that may be evaluated in this article, or claim that may be made by its manufacturer, is not guaranteed or endorsed by the publisher.

## References

[1] United Nations. World Population Prospects: The 2012 Revision. (2012).

[2] Lemon BW Begtson VL Peterson JA. An exploration of the activity theory of aging: activity types and life satisfaction among in-movers to a retirement community. J Gerontol. (1972) 27:511–23. 10.1093/geronj/27.4.511 5075497

[3] Kim JE Moen P. Retirement transitions, gender and psychological well-being: a life-course, ecological model. J Gerontol B Sci Soc Sci. (2002) 57:212–22. 10.1093/geronb/57.3.P212 11983732

[4] Menec VH. The relation between everyday activities and successful aging: a 6-year longitudinal study. J Gerontol B Sci Soc Sci. (2003) 58:S74–82. 10.1093/geronb/58.2.S74 12646596

[5] Duncan DF Whitney RJ. Work and the mental well-being of the elderly. Psychol Rep. (1990) 66:882. 10.2466/pr0.1990.66.3.882 2377707

[6] Forbes M Spence K Wuthrich VM Rapee RM. Mental health and wellbeing of older workers in Australia. Kango Japan J Nurs. (2015) 1:97–111. 10.1093/workar/wav004

[7] Havighurst RJ. Successful aging. Gerontologist. (1961) 1:8–13. 10.1093/geront/1.1.8

[8] Bender KA Jivan NA. What Makes Retirees Happy? Issue in Brief 28. Center for Retirement Research at Boston College (2005).

[9] Yanni H. Productive activities and psychological well-being among older adults. J Gerontol B Sci Soc Sci. (2008) 2:S64–72. 10.1093/geronb/63.2.S64 18441271

[10] Andiara S Niti MM Tang C Ng TP. Continued work employment and volunteerism and mental well-being of older adults: Singapore longitudinal ageing studies. Age Aging. (2009) 5:531–7. 10.1093/ageing/afp089 19474036

[11] Coe NB Zamarro G. Retirement effects on health in Europe. J Health Econ. (2011) 30:77–86. 10.1016/j.jhealeco.2010.11.002 21183235 PMC3972912

[12] Lee Y Min J Chi I. Life transitions and leisure activity engagement in later life: findings from the Consumption and Activities Mail Survey (CAMS). Aging Soc. (2017) 38:1–21. 10.1017/S0144686X17000216

[13] Liu SL Lang XJ. The impact of retirement on the physical and mental health of China's elderly population. Popul Res. (2017) 41:74–88. 33374838

[14] Deng TH He XR. The impact of retirement on the health of elderly men—an empirical study based on breakpoint regression. Popul Econ. (2016) 06:82–91.

[15] Stenholm S Vahtera J. Does retirement benefit health? Prevent Med. (2017) 100:94–295. 10.1016/j.ypmed.2017.05.007 28583661

[16] Cheng ZH Qiu HS Yu YF Li TL Wang BY. Reemployment of older adults and their (subjective) quality of life. Chin J Gerontol. (1993) 3:136–7. 6837081

[17] Huang Q Yu D. Will delaying retirement harm health? Based on the study of working after retirement. Popul. Dev. (2019) 25:76–85.

[18] Dingemans EAA. Working *After Retirement: Determinants and Consequences of Bridge Employment*. Groningen: Rijksuniversiteit Groningen (2016).

[19] Dingemans E Henkens K. Working after retirement and life satisfaction: cross-national comparative research in Europe. Res Aging. (2019) 41:648–69. 10.1177/0164027519830610 30782077

[20] Zhang QL Zhou YM. Work and happiness of the newly retired in urban China. Acta Psychol Sin. (2017) 49:472–81. 10.3724/SP.J.1041.2017.00472

[21] Xie Y Hu J. An introduction to the China family panel studies (CFPS). Chin Sociol Rev. (2014) 47:3–29. 10.2753/CSA2162-0555470101

[22] Radloff LS. The CES-D scale: a self-report depression scale for research in the general population. Appl Psychol Meas. (1977) 1:385–401. 10.1177/01466216770010030623302475

[23] Van den Broeck G Maertens M. Female employment reduces fertility in rural Senegal. PLoS ONE. (2015) 10:e122086. 10.1371/journal.pone.0122086 25816301 PMC4376695

[24] Wen ZL Ye BJ. Analyses of mediating effects: the development of methods and models. Adv Psychol Sci. (2014) 22:731–45. 10.3724/SP.J.1042.2014.00731

[25] Wang SX. Employment of the elderly in China. Popul Econ. (1990) 3:35–9.

[26] Yu L Ma LY Yin XD Fleisher B. Supported or supporting? Family structure and employment choice of the elderly in urban China. Res Labor Econ. (2016) 4:24–54.

[27] Tadd W. Aging and agism in the 21st century. Rev Clin Gerontol. (2000) 10:203–5. 10.1017/S0959259800010315

[28] Maddox GL. Disengagement theory: a critical evaluation. Gerontologist. (1964) 57:80–2. 10.1093/geront/4.2_Part_1.80 14285386

[29] Moen P. A life course perspective on retirement, gender, and well-being. J Occup Health Psychol. (1996) 1:131–44. 10.1037/1076-8998.1.2.131 9547042

[30] Carstensen LL Isaacowitz DM Charles ST. Taking time seriously: a theory of socioemotional selectivity. Amer Psychol. (1999) 54:165–81. 10.1037//0003-066X.54.3.165 10199217

[31] Van Houtven CH Coe NB Skira MM. The effect of informal care on work and wages. J Health Econ. (2013). 32:240–52. 10.1016/j.jhealeco.2012.10.006 23220459

[32] Yang J Zhang S Ning XD. Education level and post-retirement employment: evidence from CHARLS. China J Econ. (2018) 5:169–202. 10.16513/j.cnki.cje.2018.03.008

[33] Bradburn NM. The Structure of Psychological Well-Being. Chicago, IL: Aldine (1969).

